# Biotoxicity of TiO_2_ Nanoparticles on *Raphidocelis subcapitata* Microalgae Exemplified by Membrane Deformation

**DOI:** 10.3390/ijerph15030416

**Published:** 2018-02-27

**Authors:** Merve Ozkaleli, Ayca Erdem

**Affiliations:** Department of Environmental Engineering, Faculty of Engineering, Akdeniz University, Antalya 07058, Turkey; Turkeymerveozkaleli@akdeniz.edu.tr

**Keywords:** TiO_2_ nanoparticles, synthetic freshwater, membrane deformation, *Raphidocelis subcapitata*

## Abstract

TiO_2_ nanoparticles (NPs), which are mainly used in consumer products (mostly cosmetics), have been found to cause ecotoxic effects in the aquatic environment. The green algae *Raphidocelis subcapitata*, as a representative of primary producers of the freshwater ecosystem, has been frequently used to study the effects of metal oxide NPs. An ecotoxicity study was conducted herein to investigate the effects of TiO_2_ NPs on survival and membrane deformation of algal cells. Five different concentrations of nano-TiO_2_ particles (1, 10, 50, 100 and 500 mg/L) were prepared in synthetic surface water samples with five different water quality characteristics (pH 6.4–8.4, hardness 10–320 mg CaCO_3_/L, ionic strength 0.2–8 mM, and alkalinity 10–245 mg CaCO_3_/L). Results showed a significant increase in the hydrodynamic diameter of NPs with respect to both NP concentrations and ionic content of the test system. A soft synthetic freshwater system at pH 7.3 ± 0.2 appeared to provide the most effective water type, with more than 95% algal mortality observed at 50, 100 and 500 mg/L NP concentrations. At high exposure concentrations, increased malondialdehyde formations were observed. Moreover, due to membrane deformation, TEM images correlated the uptake of the NPs.

## 1. Introduction

Titanium dioxide (TiO_2_) nanoparticles (NPs) have various consumer applications in textiles, cosmetics, electronics, plastics, batteries, paints, food supplements and coatings [1,2,3,4,5,6]. Nano-sized TiO_2_ particles exhibit photoreactivity and are used as catalysts for water and waste water treatment, as well as air purification [7,8]. The application of nano-TiO_2_ is still expanding and the annual production of TiO_2_ NPs is predicted to reach 2.5 million tons by 2025 [9]. Thus, TiO_2_ NPs feature in the Organisation for Economic Co-operation and Development’s (OECD) list of manufactured nanomaterials with priority for immediate testing [10]. Uncontrollable release of TiO_2_ NPs into the receiving environment may lead to adverse effects on organisms [11,12,13,14], especially on algae. The ecotoxicity of TiO_2_ NPs to algae has been described in some studies, where nano-TiO_2_ decreased algal growth and total chlorophyll content [15,16,17,18]. In contrast, Griffitt et al. [19] showed a lower toxicity of TiO_2_ NPs to algae. However, the mechanisms of TiO_2_ NP toxicity to algae are still largely unknown.

TiO_2_ NPs can produce reactive oxygen species (ROS) and hydroxyl radicals (OH·) under UV and solar light illumination. ROS are very strong oxidants and they can decompose cell membranes [20,21]. ROS cause oxidative stress, which leads to lipid peroxidation and increased membrane deformation and cell mortality. Therefore, oxidative stress is documented as one of the main mechanisms of NP toxicity to organisms [17,22,23,24,25,26]. Some studies showed that photoreactive TiO_2_ NPs can produce oxidative stress in dark conditions [27,28,29]. Metzler et al. [16,17] observed growth inhibition of the green algae *Pseudokirchneriella subcapitata* exposed to TiO_2_ NPs under fluorescence light irradiation. ROS generation under fluorescence light causes oxidative stress on algae. Lee and An [30] showed no significant differences in the inhibition of *P. subcapitata* growth under visible light, UVA, and UVB irradiation conditions.

In this study, in order to understand the effect of TiO_2_ NPs on *Raphidocelis subcapitata*, five different concentrations of TiO_2_ NPs (1, 10, 50, 100 and 500 mg/L) were prepared in synthetic surface water samples used in an OECD algal inhibition test [31]. The objective of this paper was to determine (1) the half maximal effective concentration (EC_50_) values both in terms of “mass concentration” and “number concentration” of the TiO_2_ NPs for comparison; (2) the response of the algae to TiO_2_ NPs in terms of lipid peroxidation of the cell membrane; and (3) the transmission electron microscopy observations of the algal cells exposed to TiO_2_ NPs.

## 2. Materials and Methods

### 2.1. Algal Culture

The algal stock culture of *Raphidocelis subcapitata* (SAG61.81, Göttingen, Germany) (formerly known as *Pseudokirchneriella subcapitata* and *Selenastrum capricornutum*) was purchased from the Culture Collection of Algae, Göttingen University (SAG, Göttingen, Germany). The algae were cultured according to the OECD 201 algal growth inhibition test guidelines [31], and exponentially growing algal cultures were used. The cell density of the culture was monitored spectrophotometrically at 684 nm with a Hach DR6000 (Loughland, CO, USA) instrument and by counting with a hemocytometer every 24 h. Morphological observations were conducted with a Nikon Eclipse E100 microscope (Nikon Instruments, Melville, NY, USA).

### 2.2. Nanoparticles

Nanosized TiO_2_ was used in the experiments. TiO_2_ NPs (20–40 nm, 30–60 m^2^/g) were purchased from Alfa Aesar (Ward Hill, MA, USA). The specific surface area was obtained using an N_2_-gas adsorption analyzer (QuadraSorb Station, Quantochrome Inc., Boynton Beach, FL, USA) according to the Brunauer–Emmett–Teller (BET) method. Scanning electron microscopy (SEM, Quanta FEG 250, FEI, Hillsborough, OR, USA) and dynamic light scattering (DLS, Dynapro Nanostar, Wyatt, CA, USA) techniques, as well as energy dispersive X-ray (EDX) spectroscopy (Apollo X AMATEK) were used to determine the particle size, and to characterize the structure of the TiO_2_ NPs, respectively.

Stock suspensions of 1000 mg/L of nano-TiO_2_ were prepared in synthetic freshwater solutions (SFSs, Table 1) immediately prior to the experiments. An ultrasonic homogenizer (100 W, 20 kHz, Bandelin, Sonopuls HD2200, Berlin, Germany) equipped with a titanium probe was used for 15 min in order to prevent initial aggregation of the NPs in the stock suspensions. The final NP concentrations of 1 to 500 mg/L were prepared by serially diluting the stock suspensions. All experiments were conducted in continuously shaken aqueous slurry solutions to ensure mixing and to prevent settling of the NPs.

### 2.3. Algal Inhibition Assay

In order to determine the algal inhibition, the OECD 201: Freshwater Alga and Cyanobacteria, Growth Inhibition Test [31] was followed. All tests were conducted at 22 ± 1 °C under continuous illumination with cool white light (7.2 klux), and shaking at 100 rpm for 24, 48, and 72 h. In contrast to from the OECD guideline and according to the authors’ previous experience, a higher algal population of 2.6 ± 1.1 × 10^5^ cells/mL was harvested from a four-day stock culture, and washed and resuspended in SFSs. All experiments were repeated on three separate occasions. Growth rate inhibitions were then determined by comparison with control.

### 2.4. Lipid Peroxidation Assay

Lipid peroxidation assay described by Maness et al. [23] was applied with minor modifications. According to the method, 4 mL of the algae–NP sample was mixed with 8 mL of 10% (w/v) of trichloroacetic acid, and centrifuged for 25 min at 14,000 rpm. The supernatant was mixed with 3 mL of freshly prepared thiobarbituric acid (TBA) (0.67% w/v), boiled for 10 min, and then cooled to room temperature. The formation of pink malondialdehyde (MDA)–TBA mixture was measured using Hach-Lange Dr6000 model spectrophotometer (532 nm). The standard curve of MDA-TBA complex was used to calculate the concentration of lipid peroxidation in µM/#algae of MDA.

### 2.5. Transmission Electron Microscopy Imaging of Algae and TiO_2_ Nanoparticle Samples

TEM samples were obtained from *R. subcapitata* algal suspensions (2.6 ± 1.1 × 10^5^ algae/mL) treated with or without TiO_2_ concentrations at a concentration of 100 mg/L for 72 h. The pellet from centrifugated suspension was fixed with 4% glutaraldeyde and 0.1 M of Sorenson’s phosphate buffer (SPB) for 2 h. After washing with 0.1 M SPB (3 × 10 min), 1% osmium tetraoxide was used to fix the pellet. To wash and to dehydrate the pellets, grades (30–100%) of ethanol were used for 3 × 10 min. After infiltration of the sample with araldite resin at 23 ± 2 °C for 12 h, the sample was fixed in pure resin. The blocks were treated at 60 °C for 72 h, and ultrathin sectioning was performed using a microtome. The grids were examined under a TEM (Zeiss-Leo 906E, Carl Zeiss, Jena, Germany) operated at an accelerating voltage of 100 kV.

### 2.6. Statistical Analysis

The end point results were expressed as the mean ± SD from three independent experiments. A one-way ANOVA test (*p* < 0.05) was used to analyze the statistical significance of differences between treatment and control groups.

## 3. Results and Discussion

### 3.1. Characterization of Nanoparticles

**Figure 1:**
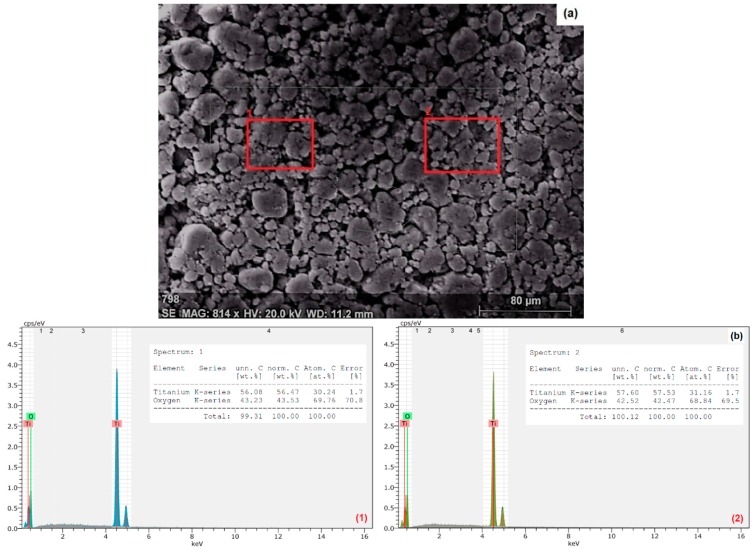
Scanning electron microscopy (SEM) (**a**) and energy dispersive X-ray (EDX) images (**b1** and **b2**) of TiO_2_ nanoparticles (concentration: 100 mg/L in ultrapure water).

The size and zeta potential of the TiO_2_ NPs in ultrapure water and in different SFSs were measured by SEM-EDX, DLS, and zeta sizer, respectively. An SEM image of the nano-TiO_2_ particles indicates a dense agglomeration and an irregularity in particle shape (Figure 1a). An average particle size from three observations was calculated as 30 ± 4.9 nm. EDX analysis was applied on two different areas (red rectangles; 1 and 2) in Figure 1a, and from the results with only Ti (56.92 ± 0.76%) and O (42.94 ± 0.53%) peaks it was shown that the sample was only composed of TiO_2_ particles (Figure 1b).

The effects of different pH and water composition on TiO_2_ NP size and zeta potentials are depicted in Table 2. The measurements were taken at time 0 and 72 h in order to correspond with the beginning and the end of the 72 h-algal inhibition test. Time 0 also shows the primary size and zeta potential value of the NPs in each of the SFSs. The primary and secondary particle sizes were measured using only DLS. Kato et al. [33,34] have shown the effectiveness of the DLS method for evaluating the particle size and stability of metal oxide NPs and culture media. An optimum NP concentration of 50 mg/L was employed in DLS measurements. It was also reported by others [35,36,37] and in our previous study [29] that at high NP concentrations, the particle size increased from one to two orders of magnitude immediately. As shown in Table 2, the pHs were depicted between 6.5 and 8.2 at time 0, and 7.0 and 8.0 at time 72 h. The ionic strength of the test solutions was from 0.5 mM to 8 mM. The primary NP size varied in the range of 20 ± 4–100 ± 49 nm, and the secondary NP sizes were between 212 ± 19 and 1428 ± 202 nm. Our results were confirmed with Leroy et al. [38] in that pH and salinity affect the stability of TiO_2_ NPs, and high ionic strength increases the size of the NPs.

The particle sizes before the exposure (*t*_0_: d_1_, primary size) and after the exposure (*t*_72 h_: d_2_, secondary size) showed a two-order polynomial correlation, as shown in Equation (1):

d_2_ = 0.0576 d_1_^2^ + 5.4655 d_1_ + 25.353 (1)

As reported by Baalousha and Lead [39], and Nur et al. [40], the high standard deviations may correspond to the polydispersity of the TiO_2_ NPs. This conclusion is also relevant to our results that the polydispersity indices of the primary and the secondary TiO_2_ NPs were of 1.03 and 1.01, respectively. The increase in ionic strengths of the SFSs (from 0.5 to 8 mM) resulted in bigger agglomerates, indicating the difficulty in obtaining primary particle size even after sonication.

Table 2 also shows the zeta potential values of TiO_2_ NPs prepared in different SFSs before and after the exposure durations. Zeta potential values were used to determine and to predict the potential and long-term stability of the NPs in test solutions. The stability of the NPs increases with greater zeta potential magnitude [36]. Before the exposure (*t*: 0), the measured zeta potential values of TiO_2_ NPs were −6.9, −16.0, −20.5, and −23 mV in very hard, moderately hard, soft, and very soft SFSs, respectively. The results indicate that at *t*: 0, NPs were moderately stable in very soft and soft SFSs, and not stable in other SFSs. After the exposure (*t*: 72 h), the zeta potential values of NPs in moderately hard—very soft SFSs increased, and those in very hard SFSs decreased. In addition, the stability of the NPs decreased. The results also showed that NPs were negatively charged in all SFSs, regardless of the water composition of the samples.

**Figure 2:**
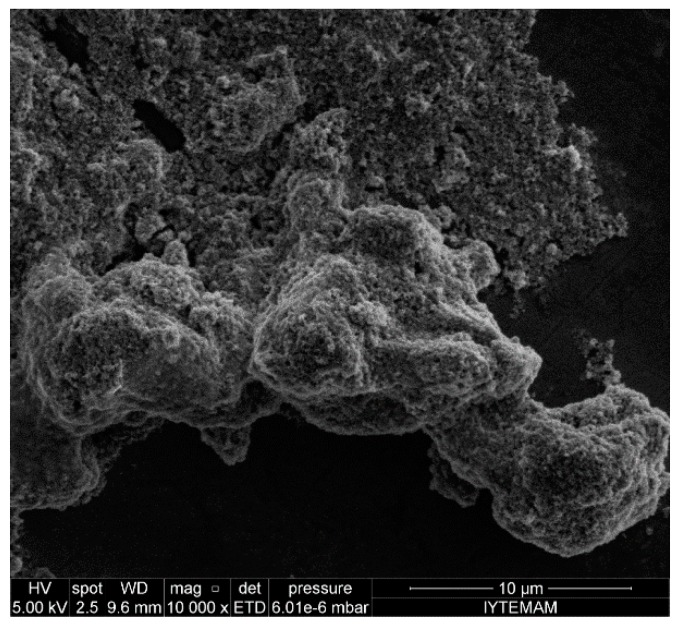
SEM image of agglomerated TiO_2_ nanoparticles in very hard synthetic surface water solution (Concentration: 100 mg/L).

Figure 2 shows different sizes of agglomerated TiO_2_ NPs prepared in very hard SFSs with a 72 h exposure time. Other studies reported the agglomeration of TiO_2_ NPs in algal medium [30,40], in plant media [40], in ultrapure water [40,41], and in water suspensions with different ionic strengths [17,27,28,36,37,40,42,43]. Lee et al. [36] reported the effect of photochemical properties and the cytotoxicity of TiO_2_ NPs depending on the degree of NP agglomeration. NP size measurements were obtained using the DLS technique and in order to stabilize the NP suspensions, NPs were stirred in phosphate buffer solution (pH: 8) for 24 h. After test duration, sonication and centrifugation steps were applied respectively to differentiate the primary (from supernatant) and aggregated NP sizes. Their results revealed that the average primary NP size was 30 ± 10 nm and the aggregated NP size was 140 ± 10 nm for 24 h in cell culture media (TiO_2_ NP conc.: 20 mg/L). Lee and An [40] studied the aggregation/agglomeration behavior of TiO_2_ (rutile) NPs in OECD algal media, ultrahigh purity water, plant media, L-variegatus media, and in media with different ionic strengths prepared with KCl and CaCl_2_. The pH, zeta potential, and hydrodynamic diameter of the NPs were measured immediately after addition of NPs and after 10 min in the media. According to their results, NPs were positively charged in all media types except in algae media, and the pHs decreased from 7.69 ± 0.36 to 6.56 ± 1.0 when NPs were added into the media. The lowest NP size was observed in ultrahigh purity water (192.1 ± 5.8 nm) and the highest NP size was in L-variegatus media (1792.0 ± 151.1 nm). Because of not exposing any organism (i.e., algae) to TiO_2_ (rutile) NPs, and with very short test durations, the NPs behaved differently compared to the TiO_2_ (anatase) NPs used in our results.

### 3.2. Algal Inhibition

**Figure 3:**
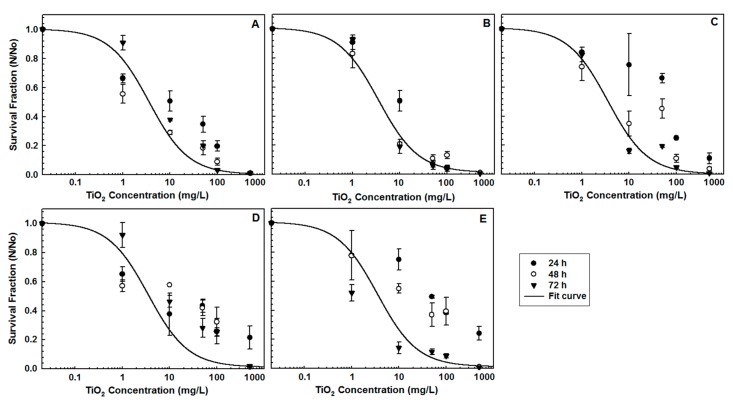
The effect of TiO_2_ nanoparticles and different water types on the survival of *Raphidocelis subcapitata* (**A**: Very soft, **B**: Soft, **C**: Moderately hard, **D**: Hard, **E**: Very hard SFSs).

In order to determine the effect of TiO_2_ NPs and different types of SFSs on *R. subcapitata*, survival fractions (N/N_0_) were calculated. Figure 3 shows the survival fractions of algae and the fitted curves of 72 h-data using a four-parameter logistic model (SigmaPlot 11.0, Starcom Info. Tech. Ltd., Bangalore, India). Regardless of the water ionic content, the increase in NP concentration led to a higher algal inhibition. However, soft SFSs at pH 7.3 ± 0.2 appeared to be the most effective water type, with more than 95% of algal mortality observed at 50, 100, and 500 mg/L NP concentrations.

The EC_50_ values were expressed in terms of mass concentration (mg/L) and number concentration (particle number/algal cell number, (*n_p_*/*n_c_*)), and the number concentration was calculated according to the following equation:

(2) $$\frac{n_{P}}{n_{c}}=\frac{3\cdot m_{P}}{4\cdot \pi \cdot \rho \cdot V\cdot C\cdot r^{3}}$$

where *n_p_* is number of TiO_2_ NPs, *mp* is mass of NPs, *r* is the radius of NPs, *ρ* is the density of TiO_2_ NPs (anatase), *n_c_* is number of algal cells, *V* is volume of sample, and *C* is concentration of the algae.

The EC_50_ values were calculated using The Toxicity Relationship Analysis Program (TRAP v1.22) (USEPA, Washington, DC, USA), and are shown in Table 3. The order of EC_50_ values from mass concentration after 72-h exposure was found to be 3.58 ± 0.16 mg/L (soft SFSs, 1 mM), 4.16 ± 0.05 mg/L (very soft SFSs, 0.5 mM), 9.32 ± 0.11 mg/L (moderately hard SFSs, 2 mM), 12.14 ± 0.09 mg/L (hard SFSs, 4 mM), and 15.57 ± 0.17 mg/L (very hard SFSs, 8 mM). According to the EC_50_ mass concentration results, soft SFSs represent the most effective water type after 72-h exposure. However, when the EC_50_ values after 24-h exposure were compared to those after 72-h exposure, no significant effect from soft and very soft SFSs were observed—these were both water types which were found to be effective for algal inhibition.

The EC_50_ values in terms of number concentration were found after 72-h exposure in an order of 5.99 ± 0.63 *n_p_*/*n_c_* (very hard SFSs), 54.5 ± 5.2 *n_p_*/*n_c_* (moderately hard SFSs), 324 ± 9.5 *n_p_*/*n_c_* (soft SFSs), and 381 ± 13.4 *n_p_*/*n_c_* (very soft SFSs). It was interesting to find that aggregation of the NPs was clearly confirmed with the results. Moreover, the increased *n_p_*/*n_c_* led to an increased surface coverage of the algal cells, inhibiting them from vital activities, i.e., photosynthesis.

It is confirmed that algal inhibition was higher in SFSs with low ionic strength (0.5–1 mM) and lower pHs (6.5–7.3) than those of other SFSs. The inhibitory effects of high NP concentrations on *R. subcapitata* were observed in other studies [16,17,19,44]. Metzler et al. [16] reported that the ecotoxic effects of TiO_2_ NPs were mainly caused by particle size and the NP–algae interaction. They also indicated that the number concentration is a better way to express the ecotoxic effect of TiO_2_ NPs on algae. Griffitt et al. [19] reported the lethal concentrations of silver, copper, aluminum, nickel, and cobalt NPs in hard water on *Danio rerio*, *Daphnia pulex*, *Ceriodaphnia dubia*, and *P. subcapitata*. The susceptibility values of *P. subcapitata* towards NPs in terms of LC50 (Lethal Concentration, 50%) were found to be 0.19 mg/L (n-Ag), 0.54 mg/L (n-Cu), 8.30 mg/L (n-Al), and 0.35 mg/L (n-Ni), and it was confirmed that *P. subcapitata* was the most sensitive organism to Ni NPs. Fekete-Kertész et al. [45] showed a 32–50% inhibition of *P. subcapitata* when organisms were treated with 3.13–25 mg/L TiO_2_ NP concentrations, and a greater NP toxicity effect caused by high NP concentration of 50 mg/L. The effect of two TiO_2_ NPs (anatase and rutile) on the growth of *P. subcapitata* using different exposure systems was reported by Manier et al. [46]. The EC_50_ values were obtained as 8.5 mg/L from 24-well microplate system, 2.7 mg/L from cylindrical vial system, and >50 mg/L from the erlenmeyer flask system.

### 3.3. Lipid Peroxidation: MDA Formation

**Figure 4:**
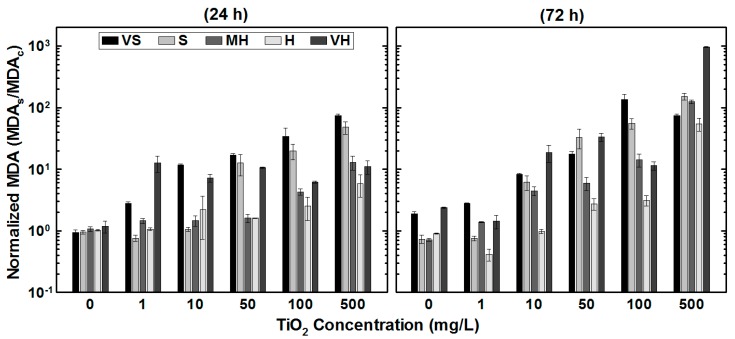
The effect of TiO_2_ nanoparticles on malondialdehyde production under the influence of different water types

The cell membrane deformation in terms of normalized MDA results is depicted in Figure 4. Normalized MDA results were obtained by dividing MDA formation value of the sample by the control’s MDA value. Within the 24-h test duration, the maximum MDA production values of *R. subcapitata* increased from 0.2 ± 0.012 µM/#algae (1 mg/L NP concentration, very soft SFSs) to 11.2 ± 0.3 πM/#algae (500 mg/L NP concentration, soft SFSs). At the end of the 72-h exposure, the maximum MDA production increased from 0.3 ± 0.009 µM/#algae (1 mg/L NP concentration, very soft SFSs) to 122.6 ± 11.5 µM/#algae (500 mg/L NP concentration, very hard SFSs). As depicted in Figure 4, highest MDA productions were observed at high NP concentration regardless of the water type. Comparing the results of normalized MDA values of the control to those of at 500 mg/L NP concentration over a 72-h test duration, 100× more MDA was produced in very hard SFSs. Results implied that the produced ROS increased the vulnerability of the cell membrane, which also shown in the TEM images.

An increase in MDA content was also shown in our previous studies [16,17] and in other studies [47,48,49,50,51,52]. Li et al. [47] showed that MDA content of *Pavlova viridis* was increased significantly from 0.5 mg/L of copper and 3.25 mg/L of zinc. Soto et al. [48] evaluated the effect of copper and zinc on *P. subcapitata* lipid peroxidation using thiobarbituric acid reactive substances assay and reported a significant malondialdehyde increase at 0.025 mg/L copper and 0.1 mg/L zinc. Li et al. [49] found that 1.0 mg/L of Al_2_O_3_ NPs had a less effect on MDA production, whereas at high copper concentrations, high MDA productions were measured from both Cu and Cu + Al_2_O_3_ exposure systems. Srivastava et al. [50] reported an increase in the content of MDA when *Hydrilla verticillata* exposed to copper. Suman et al. [51] found an increase in MDA production from the marine algae *Chlorella vulgaris* as the dose of ZnO NPs increased. Chen et al. [52] showed that MDA content of *Chlamydomonas reinhardtii* reached maximum values of 0.18 µmol/mg after 8-h exposure and then decreased to 0.04 µmol/mg at 72 h.

### 3.4. TEM Imaging

**Figure 5:**
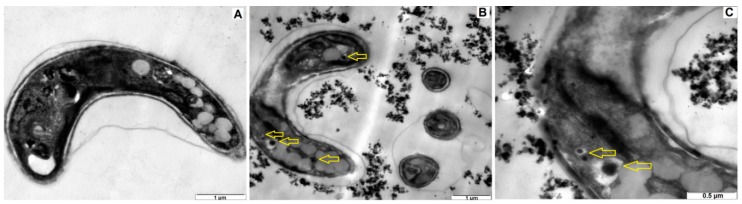
Transmission Electron Microscopy (TEM) images of healthy algae (**A**) and algae + TiO_2_ nanoparticles (**B**,**C**). (TiO_2_ concentration: 100 mg/L, water type: moderately hard synthetic freshwater solutions, T: 21 ± 2 °C, and pH: 7.6).

Figure 5 shows the TEM images of healthy algae (Figure 5A) and algae exposed to TiO_2_ NPs (Figure 5B,C). A TiO_2_ NP concentration of 100 mg/L was used in moderately hard SFSs at pH: 7.6. Aggregated and single TiO_2_ NPs were observed around and attached to the algae. Similar findings were also reported in other studies [16,52], and NP–NP and algae–NP interactions were depicted. Moreover, according to Metzler et al. [16] the pH of zero point of charge (pH_ZPC_) and the pH of TiO_2_ NPs were within the range of 7.5 to 8.0, and algae were negatively charged in the test chambers. Electrostatic force played an important role in the NP–algae aggregation. Aruoja et al. [53] showed that *P. subcapitata* can adsorb 2.3 times its own weight of TiO_2_ NPs at pH 5.5. TEM images also show that the cell integrity was compromised and permeability of the cell membrane was visible. The images also clearly express an uptake of NPs through cell membrane.

Internalization of NPs is another possible pathway apart from cell membrane damage. The kinetics of internalization might be result of diffusion flux from the NP solution to the surface of algae. Even though the adsorption is faster than the mass transfer, diffusion flux triggers the bioavailability of NPs. Bioavailability of NPs mainly depends on the number and/or mass concentration, the surface properties of NPs, and the membrane structure of cells. Nano-Ag particle internalization into *Ochromonas danica* with endocytosis ability [54], and TiO_2_ NP internalization into *Anabaena variabilis* [55] have been reported. Ekstrand-Hammarström et al. [56] and Nur et al. [40] showed that the bioavailability of NPs can be determined via their aggregation behavior, which plays an important role defining in abiotic (NP–NP) and biotic (NP–algae, especially internalization of NPs) relationships in ecotoxicity tests. Sendra et al. [57] showed a higher ionic strength can inhibit the internalization processes of TiO_2_ NPs with the freshwater algae *Chlamydomonas reinhardtii* and marine algae *Phaeodactylum tricornutum.* Metzler et al. [16] confirmed the aggregation and adsorption of TiO_2_ NPs at 100 and 250 μg/mL with *P. subcapitata*, and also revealed DNA leakage from algal cells.

## 4. Conclusions

Microalgae, as primary producers in aquatic environments, can be used as indicator organisms (biomarkers) in evaluating the aquatic toxicity of potential pollutants. Among those pollutants, NPs may drastically change ecosystems, leading to modifications in the food chain. With increasing use of NPs, their entry into the freshwater environment is inevitable. Therefore, it is extremely relevant to employ microalgae in ecotoxicity studies. The present study demonstrated the ecotoxic effects of TiO_2_ NPs on *R. subcapitata*. Our results show that TiO_2_ NPs inhibit the growth of *R. subcapitata* under visible light conditions, and show for the first time that different water content may also play a significant role in the toxicity of TiO_2_ NPs. TiO_2_ NPs increase the lipid peroxidation of the cell membrane, resulting in the deformation of the membrane structure. Even though aggregation of NPs was observed, an uptake of NPs into the algae was also confirmed. Nevertheless, the understanding of membrane deformation and TiO_2_ NP uptake mechanisms is still limited with the current state of information. Therefore, further research should be implemented to increase current knowledge on the ecotoxicity of TiO_2_ and other metal oxides.

## Figures and Tables

**Table 1:** Preparation of synthetic freshwater using reagent-grade chemicals [32].

Water Type	Reagents Added to Deionized Water (mg/L)				Approximate Final Water Quality		
	NaHCO_3_	CaSO_4_·H_2_O	MgSO_4_	KCl	pH ^1^	Hardness ^2^	Alkalinity ^2^
Very soft	12.0	7.5	7.5	0.5	6.4–6.8	10–13	10–13
Soft	48.0	30.0	30.0	2.0	7.2–7.6	40–48	30–35
Moderately hard	96.0	60.0	60.0	4.0	7.4–7.8	80–100	57–64
Hard	192.0	120.0	120.0	8.0	7.6–7.8	160–180	110–120
Very hard	384.0	240.0	240.0	16.0	8.0–8.4	280–320	225–245

^1^ pH equilibrium after 24 h, ^2^ mg CaCO_3_ L^−1^.

**Table 2:** The effect of pH and synthetic freshwater solution type on TiO_2_ nanoparticle size and zeta potential. (Nanoparticle concentration: 50 mg/L).

Synthetic Freshwater Solution Type	Ionic Strength (mM)	pH		Nanoparticle Size (nm)		Zeta Potential (mV)	
		0	72 h	0	72 h	0	72 h
Very soft	0.5	6.5 ± 0.3	7.0 ± 0.2	20 ± 4	212 ± 19	−23.0	−18.4
Soft	1	7.3 ± 0.2	7.5 ± 0.1	44 ± 7	287 ± 25	−20.5	−17.6
Moderately hard	2	7.6 ± 0.1	7.8 ± 0.2	61 ± 22	546 ± 71	−16.0	−15.7
Very hard	8	8.2 ± 0.2	8.0 ± 0.2	100 ± 49	1428 ± 202	−6.9	−13.4

**Table 3:** The half maximal effective concentration (EC_50_) values in terms of mass concentration (mg/L) and number concentration (*n_p_*/*n_c_*).

Synthetic Freshwater Solution Type	Mass Concentration (mg/L)		Number Concentration (*n_p_*/*n_c_*)	
	24 h	72 h	24 h	72 h
Very soft	18.12 ± 1.3	4.16 ± 0.05	1698 ± 214	381 ± 13.4
Soft	17.96 ± 2.8	3.58 ± 0.16	645 ± 98	324 ± 9.5
Moderately hard	56.4 ± 9.9	9.32 ± 0.11	60.3 ± 24	54.5 ± 5.2
Very hard	98.3 ± 12.4	12.14 ± 0.09	16.2 ± 3.5	5.99 ± 0.63
